# Quantum vibropolaritonic sensing

**DOI:** 10.1126/sciadv.ady7670

**Published:** 2025-08-15

**Authors:** Peng Zheng, Steve Semancik, Ishan Barman

**Affiliations:** ^1^ Department of Mechanical Engineering, Johns Hopkins University, Baltimore, MD 21218, USA.; ^2^ Biomolecular Measurement Division, Material Measurement Laboratory, National Institute of Standards and Technology, Gaithersburg, MD 20899, USA.; ^3^ Department of Oncology, Johns Hopkins University School of Medicine, Baltimore, MD 21287, USA.; ^4^ The Russell H. Morgan Department of Radiology and Radiological Science, Johns Hopkins University School of Medicine, Baltimore, MD 21287, USA.

## Abstract

Vibrational spectroscopies are pivotal in analytical methods and biomedical diagnostics owing to their singular ability to provide molecular specificity. However, they are intrinsically limited by weak light-matter interactions and vulnerability to intensity fluctuations and spectral interference. Here, we propose a quantum sensing strategy by leveraging hybrid light-matter states under vibrational strong coupling between molecular vibrations and an optical cavity mode. These quantum vibropolaritonic states exhibit characteristic vacuum Rabi splitting, which not only enables manipulation of molecular vibrations but also provides a unique optical transducer. The feasibility of this strategy is established by combining theoretical analysis and numerical simulations. Through fabrication of a microfluidic infrared flow cell, definitive experimental validation of vibropolaritonic sensing is achieved. We believe that this study represents a major advance in harnessing hybrid light-matter states for molecular sensing and offers exciting potential to affect applications in areas including chemical sensing, environmental monitoring, biomedical diagnostics, and bioprocess monitoring.

## Teaser

Hybrid light-matter states in the form of vibropolaritons are harnessed to develop quantum-enabled vibrational spectroscopy.

## INTRODUCTION

Vibrational spectroscopies, as exemplified by infrared (IR) and Raman spectroscopies, have long been a cornerstone in analytical and diagnostic sectors due to their high molecular specificity (*1*–*4*). By probing molecular vibrational transitions, these techniques offer detailed chemical fingerprints for rapid identification of molecular structures and compositions. As a result, they find a wide range of application in areas including biopharmaceuticals, biomedicine, environmental monitoring, food safety, and forensic sciences (*5*–*9*). However, conventional vibrational spectroscopies are inherently limited by the weak-coupling nature of light-matter interactions. In this weak-coupling regime, the coupling strength is not strong enough to compensate for energy losses. Consequently, molecular vibrations are passively perturbed and probed by the used incident light source. Furthermore, the transducing mechanisms in these spectroscopies mainly rely on changes in intensity or one-way frequency shifts (*10*–*13*). The transduced intensity and frequency-shift signals are susceptible to intrinsic intensity fluctuations and can be masked by subtle spectral variations, thereby compromising sensing performance (*14*–*16*). Additional challenges such as background interference, thermal fluctuations, and spectral congestion further complicate identification of molecular signatures, undermining the specificity and reliability of these techniques (*17*, *18*). These limitations underscore the unmet need for advanced sensing mechanisms which not only probe molecular vibrations, but also actively enhance and manipulate their vibrational spectral features.

Molecular vibrations, which underpin vibrational spectroscopies and arise from quantized energy levels within molecules, are inherently quantum mechanical in nature (*19*–*22*). Exploring quantum aspects of molecular vibrations could provide unique approaches to augmenting the capabilities of vibrational spectroscopies. However, the tantalizing possibility of leveraging these quantum phenomena for advanced vibrational spectroscopies remains largely untapped.

Recently, we introduced an innovative optical sensing strategy called quantum plexcitonic sensing by leveraging hybrid light-matter states formed through coherent plasmon-exciton interactions (*23*). This approach transduces an optical signal based on the vacuum Rabi splitting and enables ultrasensitive, robust optical sensing even under the influence of optical noise. Building on this concept, we aim to extend the strategy through exploration of vibrational strong coupling (VSC), where molecular vibrations are strongly coupled with a cavity mode of the quantized radiation field. Under VSC, the coherent interaction between the cavity and molecular vibrations produces a pair of quantum vibropolaritonic states, which are spectrally manifested as vacuum Rabi splitting. By leveraging these vibropolaritonic states, VSC opens a fundamentally unique approach for manipulating and probing molecular vibrations (*24*–*28*). To overcome limitations posed by the intrinsically weak transition dipole moments of most molecules, we aim to use ensemble strong coupling (*29*, *30*) to achieve VSC, where a large number of molecular vibrations are collectively coupled to a single cavity mode. The concept of ensemble strong coupling, originally proposed in 1984 by Agarwal (*31*), laid the foundation for achieving hybrid light-matter states in molecular systems. Despite the potential of ensemble strong coupling for developing quantum optical sensing platforms, its application in molecular sensing to date has remained largely unexplored.

In this study, we present a systematic investigation of quantum vibropolaritonic sensing, which includes theoretical analyses combined with finite-difference time-domain (FDTD) numerical simulations and proof-of-concept experimental demonstrations. We use a Fabry-Pérot (FP) cavity consisting of gold mirrors on CaF_2_ IR windows as a model platform, where molecular analytes in solution are introduced to achieve VSC. The analytes are modeled as Lorentz oscillators, and the effective relative permittivity is calculated using the volume fraction–weighted mixing rule. Our FDTD simulations demonstrate the formation of quantum vibropolaritonic states, which are characterized by energy-level anticrossing behavior (*32*–*34*). We also conducted concentration-dependent and noise-modulated studies to demonstrate the feasibility and robustness of the approach. Last, a microfluidic IR flow cell with various cavity lengths was used to provide experimental validation and showcase the potential of quantum vibropolaritonic sensing.

## RESULTS

### Principle of quantum vibropolaritonic sensing

**Fig. 1.**
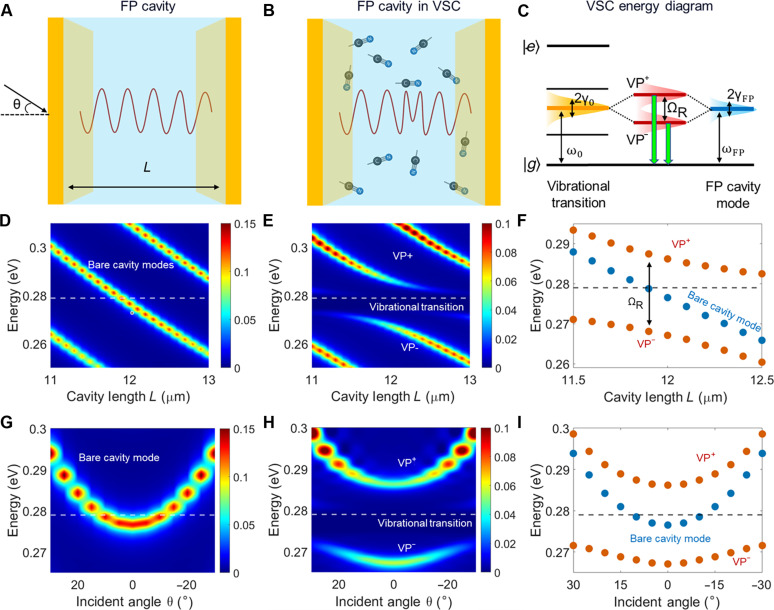
Principle of quantum vibropolaritonic sensing. Schematics of (**A**) an FP cavity, which is uncoupled in air, and (**B**) an FP cavity in VSC with the enclosed molecular analytes in solution. In (A) and (B), the FP cavity is made of a pair of gold mirrors with a cavity length of *L* and an incident light angle of θ. (**C**) VSC energy diagram, where the molecular vibrational transition has a frequency of $\omega_{0}$ and a linewidth of $2\gamma_{0}$ , while the FP cavity mode has a resonant frequency of $\omega_{\text{FP}}$ and a linewidth of $2\gamma_{\text{FP}}$ . (**D**) Cavity length *L*–dependent and (**G**) incident angle θ–dependent evolution of FP cavity modes. VSC anticrossing behavior observed through modulating (**E**) the cavity length *L* and (**H**) the incident angle θ. Comparisons of VSC with an FP cavity mode and vibrational transition line for (**F**) cavity length *L*–dependent modulation and (**I**) incident angle θ–dependent modulation. The data presented in (D) to (I) were calculated on the basis of theoretical analysis and FDTD simulations; details can be found in Materials and Methods. The gold mirrors of the FP cavities are modeled to have a thickness of 10 nm. For (D) to (I), the oscillator strength *F* of the MBN molecular analyte is fixed at 0.01; for (G) to (I), the cavity length is fixed at *L* = 12 μm. $\Omega_{R}$ stands for vacuum Rabi splitting.

We propose a general quantum vibropolaritonic sensing strategy for detecting molecular analytes in solution by leveraging ensemble strong coupling, where *N* analyte molecules collectively couple with a single mode of the quantized radiation field (*29*, *30*). To demonstrate this concept, we selected 4-mercaptobenzonitrile (4-MBN) as the target analyte and used an FP cavity with a cavity length of *L* as the sensing platform, as schematically illustrated in Fig. 1 (A and B). As a proof of concept, we targeted the vibrational transition of the nitrile group (─C≡N) in MBN molecules, which occurs at ~2250 cm^−1^. This specific transition frequency falls within the IR transparent window, a spectral region largely free of strong molecular absorption bands. The absence of background interference in this region makes it an ideal choice for enhancing spectral contrast and minimizing unwanted absorption from solvents or other molecular species that could otherwise obscure vibropolaritonic spectral features. Under VSC, quantum vibropolaritonic states are expected to form under ambient conditions, as illustrated in Fig. 1C. They are manifested as a pair of spectrally measurable peaks, which are separated by an analyte concentration–dependent vacuum Rabi splitting $\Omega_{R}$ . This analyte concentration–dependent Rabi splitting provides a direct method for quantifying molecular concentration, forming the basis for quantitative quantum vibropolaritonic sensing.

As the first step, we performed theoretical analysis in combination with FDTD numerical simulations to elucidate key features of quantum vibropolaritonic states. For the devised experimental demonstrations, we considered that MBN molecules were dissolved in dimethyl sulfoxide (DMSO), forming sample medium with a series of concentrations. While the nitrile transition dipole moment of a single MBN molecule ( μ≈0.1D ) is too weak to strongly interact with the FP cavity mode, an ensemble of MBN molecules in DMSO could collectively couple with an FP cavity mode and achieve VSC. To understand how the effective optical property of MBN molecules in DMSO depends on its concentration, we modeled the MBN molecule as a Lorentz oscillator (*35*, *36*). We further used the volume fraction–weighted mixing rule (*37*, *38*) to model its effective relative permittivity. In this study, the relative permittivity of DMSO was fixed at a constant value of 2.13. Details of the theoretical analysis can be found in Materials and Methods. The obtained concentration-dependent effective relative permittivity of MBN in DMSO is given by the following explicit form

$$\varepsilon_{e}=2.13+\frac{{\left(0.83C\right)}^{2}\cdot \omega_{0}^{2}}{\omega_{0}^{2}-\omega^{2}-i\gamma_{0}\omega }$$ (1)

where the MBN molar concentration C takes the unit of millimolars and $\omega_{0}$ is its vibrational transition energy with a damping rate of $\gamma_{0}$ with a unit of electron volts. Equation 1 suggests that the effective relative permittivity of MBN in DMSO behaves similar to a Lorentz oscillator with a high-frequency permittivity of 2.13, where the effective oscillator strength $F={\left(0.83C\right)}^{2}$ strongly depends on the analyte’s molar concentration. On the basis of Eq. 1, we used FDTD numerical simulations to investigate VSC between an FP cavity and the enclosed MBN in DMSO. Compared to the calculated FP cavity modes (Fig. 1, D and G), the calculated transmission spectra revealed a distinct anticrossing behavior of the two vibropolaritonic branches (VP^+^ and VP^−^) by tuning either the cavity length *L* (Fig. 1, E and F) or the incident angle θ (Fig. 1, H and I). This anticrossing behavior (*32*–*34*) is a hallmark of strong coupling between molecular vibrations and the quantized cavity field, demonstrating the theoretical realization of quantum vibropolaritonic states. To be consistent with the energy diagram depicted in Fig. 1C, we displayed the Fourier transform IR (FTIR) spectra with the energy unit (in electron volts) rather than the typical wave number unit (per centimeter). This also facilitates visualization of the extent of energy-level splitting.

### Theoretical detection limit of quantum vibropolaritonic sensing

**Fig. 2.**
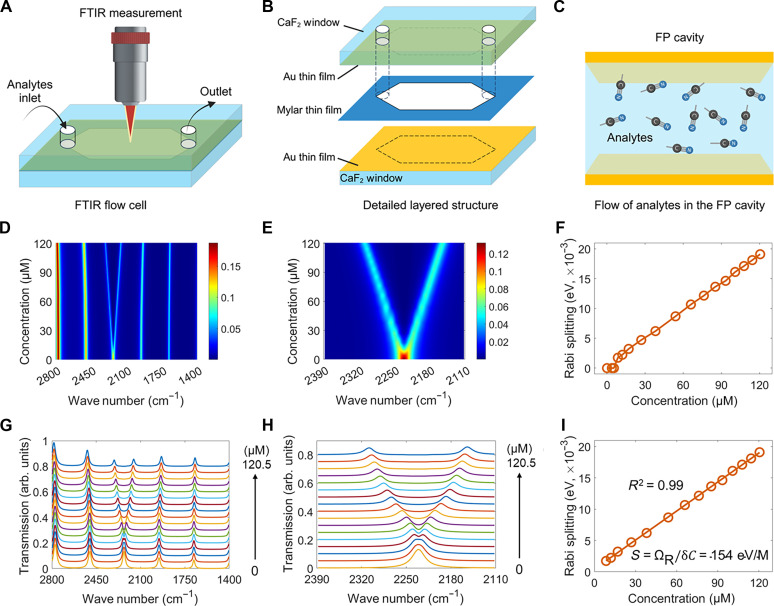
Theoretical analysis of quantum vibropolaritonic sensing. Schematics of (**A**) the FTIR flow cell for quantum vibropolaritonic sensing, (**B**) decomposition of each part of the FTIR flow cell, and (**C**) flow of the enclosed analytes in a solvent within an FP cavity. (**D** and **E** and **G** and **H**) Calculated FTIR spectra using FDTD numerical simulations (details can be found in Materials and Methods) for the modeled analytes strongly coupled with the FP cavity. arb. units, arbitrary units. (**F**) The increase in Rabi splitting with respect to increasing concentration of the modeled analytes. (**I**) Linear regression analysis of the correlation between Rabi splitting and the modeled analyte concentration. The gold mirrors of the FP cavities are modeled to have a thickness of 10 nm and a cavity length of 12 μm. The analytes are modeled to have a vibrational transition near 2250 cm^−1^. In (D) and (E), the color bar is unitless and represents the fraction of incident light that passes through the sample. For the spectra presented in (G) and (H), which have been arbitrarily offset for clarity, from bottom to top, the concentrations are (0, 3.8, 5.4, 8.5, 12.0, 17.0, 26.9, 38.1, 53.9, 66.0, 76.2, 85.2, 93.3, 100.8, 107.8, 114.3, and 120.5) µmol/L. In (G) and (H), varying colors are used for clarity. (F) and (I) differ in that the transition from no splitting to Rabi splitting at around 8.5 µmol/L was presented in (F).

Building on Eq. 1 and the successful realization of quantum vibropolaritonic states represented in Fig. 1, we proceeded to evaluate the theoretical detection limit using FDTD simulations. Toward this goal, we conceptualized an FTIR microfluidic flow cell with an inlet and outlet, as illustrated in Fig. 2 (A to C), which is consistent with the physical microfluidic device we developed for experimental demonstrations. Under VSC, by varying MBN concentrations, we observe the emergence of Rabi splitting, which displays progressively larger energy splitting for increasing MBN concentration, as shown in Fig. 2 (D, E, G, and H). By plotting Rabi splitting in relation to the MBN concentration, we determined the Rabi splitting threshold occurs at about 8.5 µmol/L (Fig. 2F). Above this threshold, the Rabi splitting energy displays a linear correlation with the MBN concentration (Fig. 2I). Together, the results shown in Fig. 2 establish the feasibility of quantum vibropolaritonic sensing, while also suggesting that the potential detection limit owing to the stringent condition of ensemble VSC is capped at about 10 µmol/L (unless additional enhancement mechanisms are used). Nonetheless, the quantum vibropolaritonic states provide an exquisite strategy for molecular sensing by manipulating molecular energy levels through light-matter hybridization.

### Noise-modulated quantum vibropolaritonic sensing

**Fig. 3.**
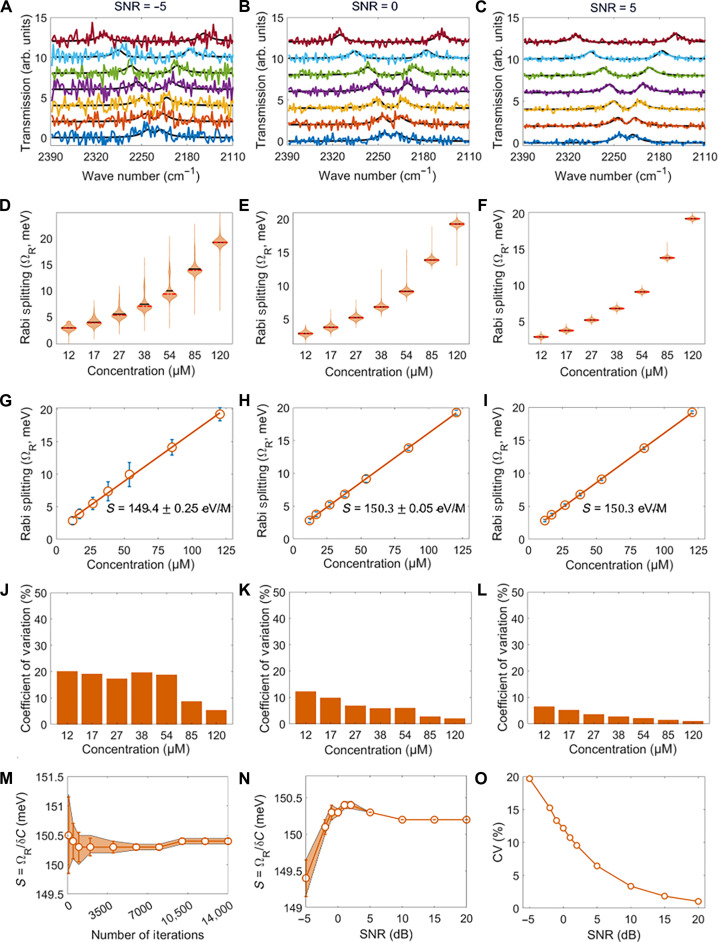
Noise-modulated quantum vibropolaritonic sensing via Monte Carlo simulations. White noise with an SNR of (**A**) −5 dB, (**B**) 0 dB, and (**C**) 5 dB was respectively added to the FTIR spectra in Fig. 2H. (**D** to **F**) Violin plots, (**G** to **I**) linear regression analysis, and (**J** to **L**) concentration-dependent coefficients of variation (CVs), obtained on the basis of the noisy spectra in (A) to (C), respectively. (**M**) Number of iteration-dependent sensitivity. (**N**) SNR-dependent sensitivity. (**O**) SNR-dependent CV for an analyte concentration of 12.0 µmol/L. For the spectra presented in (A) to (C), which have been arbitrarily offset for clarity, from bottom to top, the concentrations are (12.0, 17.0, 26.9, 38.1, 53.9, 85.2, and 120.5) µmol/L.

In practical applications, optical noise could mask vibropolaritonic spectral features, potentially compromising the platform’s sensing performance. To evaluate the robustness of quantum vibropolaritonic sensing under the influence of optical noise, following approaches from previous studies (*23*, *39*–*41*), we added artificial white noise with varying signal-to-noise ratio (SNR) levels to the selected transmission spectra. The white noise was modeled as the additive white Gaussian noise (AWGN) (*41*–*43*), which is independent of the light frequency and primarily contributed by ambient light sources. Representative FTIR transmission spectra with AWGN of varying SNR levels are presented in Fig. 3 (A to C). As expected, for a decreasing SNR, the spectra become noisier. However, because of the stochastic nature of AWGN, the noise pattern fluctuates with each simulation iteration, even when the same SNR is applied. This intrinsic randomness introduces variability in the resulting spectral profiles, which could lead to sensitivity variations, particularly at lower SNR levels.

To systematically investigate variability in noise-induced sensitivity, we used Monte Carlo simulations. By repeatedly adding AWGN to the spectra across 13,000 iterations, we ensured statistical robustness in our analysis. Such an optimized iteration number was determined on the basis of the assessment of the convergence behavior of the sensitivity shown in Fig. 3M. Following noise addition, Lorentzian fitting was applied to extract the Rabi splitting energies. The results are presented as violin plots to illustrate the distribution of Rabi splitting across various analyte concentrations and with different SNRs in Fig. 3 (D to F). Notably, while decreased SNRs broaden the distribution to a certain extent, the underlying linear relationships between Rabi splitting and analyte concentration remain largely consistent, as reflected in the strong linear correlations shown in Fig. 3 (G to I).

To further assess the sensitivity stability, we evaluated the coefficient of variation (CV) across various SNRs and different analyte concentrations (Fig. 3, J to L). The CV data reveal that the variability decreases at higher analyte concentrations and becomes smaller for larger SNRs. Even at the lowest tested SNR (−5 dB), CV values remained within 20%, underscoring the platform’s robustness. By calculating the SNR-dependent sensitivity (Fig. 3N) and the SNR-dependent CV for various analyte concentrations (Fig. 3O and fig. S1), we further established that quantum vibropolaritonic sensing maintains a robust sensitivity and precision, even under the influence of optical noise.

### Experimental realization and modulation of vibropolaritons

**Fig. 4.**
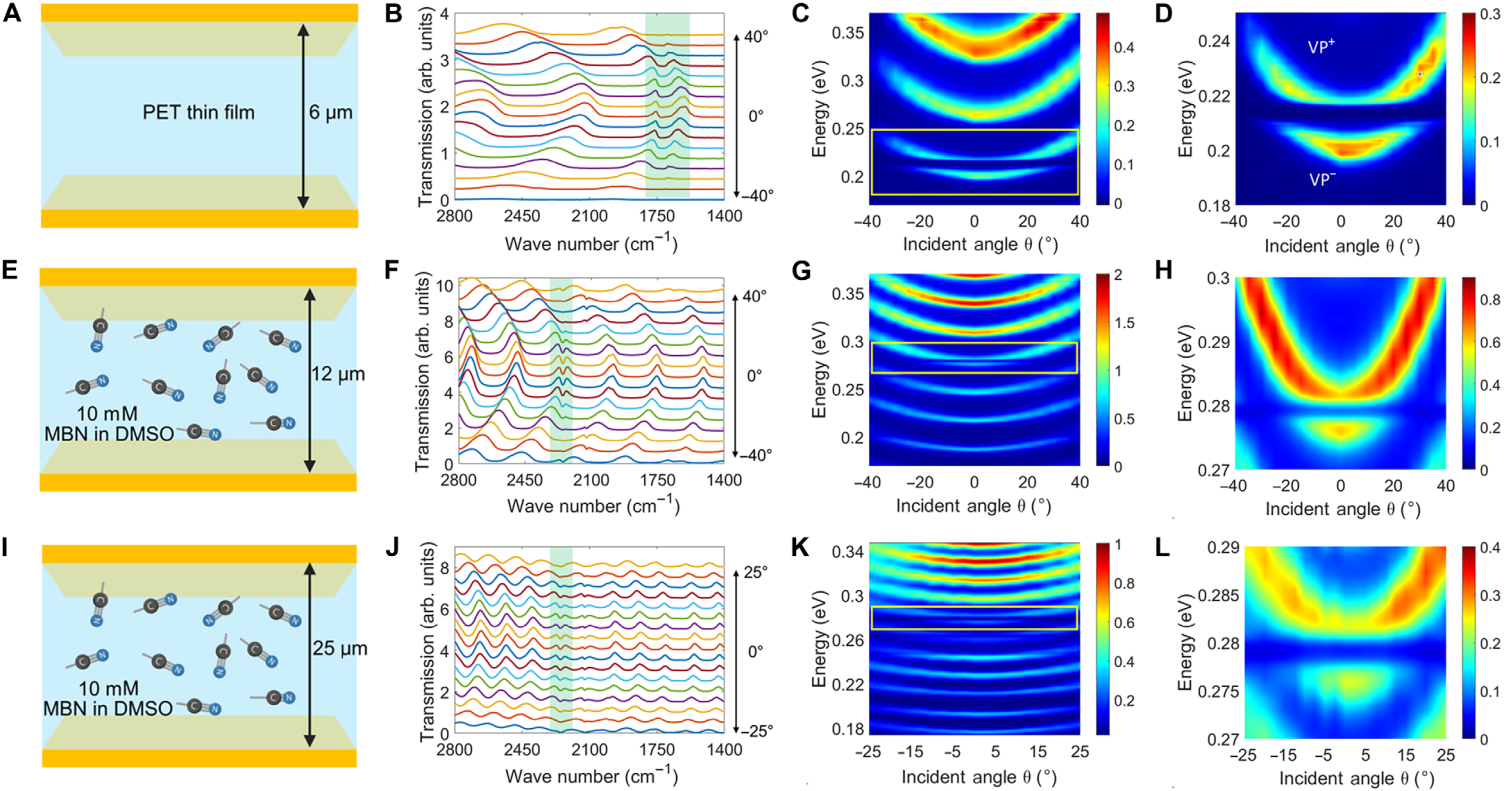
Experimental realization and modulation of vibropolaritons. Schematics of (**A**) an FP cavity with a cavity length of 6 μm enclosing a PET thin film, (**E**) an FP cavity with a cavity length of 12 μm enclosing 10 mmol/L MBN in DMSO, and (**I**) an FP cavity with a cavity length of 25 μm enclosing 10 mmol/L MBN in DMSO. The incident angle θ–dependent FTIR spectra presented in (**B**) to (**D**), (**F**) to (**H**), and (**J**) to (**L**) were measured on the basis of the configurations in (A), (E), and (I), respectively, where Rabi splitting was observed for different combinations of molecular analytes and FP cavity lengths. In (A), (E), and (I), the gold mirrors of the FP cavities have a thickness of 12 nm; the IR windows are made of CaF_2_. In (C), (D), (G), (H), (K), and (L), the color bar has a unit of percentage (%), which represents the fraction of incident light that passes through the sample. In (B), (F), and (J), the collected transmission spectra, which have been arbitrarily offset for clarity, are dominated by different orders of the FP cavity resonance modes except for the green-highlighted region where VSC occurs. Although (E) and (I) have a different cavity length, the associated FP cavities can be tuned to support vibropolaritonic states at the same wavenumber, highlighting their versatility.

Following theoretical and numerical studies, which have been discussed above, we proceeded to demonstrate quantum vibropolaritonic sensing experimentally. We began by establishing the feasibility of achieving quantum vibropolaritonic states through VSC between an FP cavity and the enclosed molecular analytes. To achieve this, we fabricated FP cavities by assembling a pair of gold mirrors into the Specac Omni Cell demountable cell with the desired spacer thickness (details can be found in Materials and Methods). Each of the gold mirrors has 12 nm of Au deposited on a CaF_2_ IR window. The FP cavity lengths were carefully adjusted to ensure that each FP cavity mode is in resonance with the vibrational transition of the specific analytes being studied. In our initial experiment, we assembled an FP cavity with a nominal cavity length of ~6 μm, which enclosed a polyethylene terephthalate (PET) thin film in air. By fine-tuning the cavity length to produce resonance at the PET’s prominent carbonyl (C═O) stretching mode near 1725 cm^−1^, we successfully achieved VSC. Incident angle-resolved FTIR measurements revealed the hallmark energy-level anticrossing behavior shown in Fig. 4 (A to D), which confirms the formation of vibropolaritonic states. This observation provided clear experimental evidence of successful VSC in a solid-state molecular system. Compared to conventional FTIR transmission measurements, FP cavities often display a much smaller transmission efficiency owing to the low transmittance of gold film in the IR. Nonetheless, vibropolaritonic states can be clearly measured and discerned.

To extend this concept to solution-based molecular systems, we experimentally fabricated a microfluidic FTIR flow cell as described in Fig. 2 (A to C). Using this setup, we introduced 10 mmol/L solutions of MBN in DMSO into FP cavities with nominal cavity lengths of 12 and 25 μm, respectively. By fine-tuning the cavity length in each case, we successfully achieved VSC in both configurations. The corresponding FTIR spectra revealed distinct anticrossing features, as illustrated in Fig. 4 (E to H and I to L), further confirming the formation of vibropolaritonic states in this solution.

### Experimental demonstration of quantum vibropolaritonic sensing

Building on the successful experimental realization of quantum vibropolaritonic states, we further investigated the potential of using these hybrid light-matter states as a quantum transducer by modulating the coupling strength using systematic variations in analyte concentration. We conducted this study based on the same FP cavity design represented schematically in Fig. 2 (A to C); the experimentally assembled cavity is depicted in Fig. 4E. The FP cavity was fine-tuned by adjusting the screws of the flow device (see more in Materials and Methods) to be in resonance with the nitrile vibrational transition of MBN in DMSO at ~2250 cm^−1^.

**Fig. 5.**
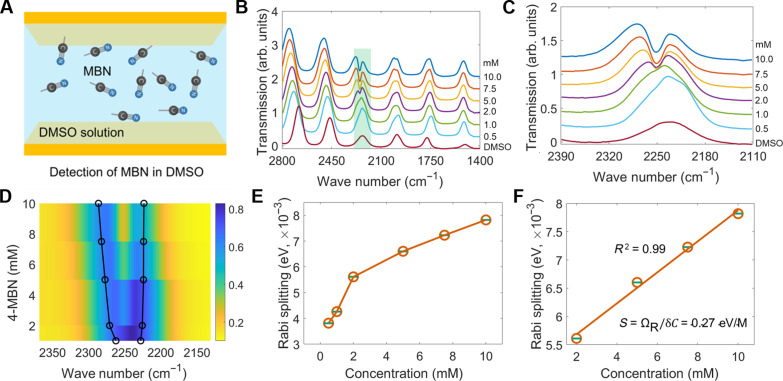
Experimental demonstration of quantum vibropolaritonic sensing. (**A**) Schematic of detection of MBN in DMSO in an FP cavity made of a pair of gold films on CaF_2_ window with a cavity length of 12 μm. (**B**) Transmission spectral evolution with increasing MBN concentration. (**C** and **D**) Observation of Rabi splitting. (**E**) Increase in Rabi splitting in relation to the MBN concentration. (**F**) Linear regression analysis of the correlation between Rabi splitting and the MBN concentration. For each concentration, the measurements were repeated three times, which returned largely similar spectra and small error bars (green) as shown in (E) and (F). The error bars represent ±1 SD for three measurements. In (B) and (C), the transmission spectra have been arbitrarily offset for clarity. In (D), the color bar has a unit of percentage (%), which represents the fraction of incident light that passes through the sample.

To evaluate the concentration-dependent modulation of vibropolaritonic coupling, we introduced MBN analytes dissolved in DMSO at varying concentrations into the microfluidic FTIR flow cell, as illustrated in Fig. 5A. The experimental design facilitated injecting the analyte solution to the cavity region and allowed precise control over the concentration of the analyte. The collected transmission spectra at lower MBN concentrations were dominated by different orders of the FP cavity resonance modes without vibropolaritonic spectral features (Fig. 5B). At higher concentrations, we observed the emergence of characteristic Rabi splitting, which is the hallmark of VSC (Fig. 5, B to D). Notably, the onset of Rabi splitting was first observed at an MBN concentration of 2 mmol/L (Fig. 5, B to E), which is approximately three times lower than the minimum concentration required for detecting the nitrile absorption peak in MBN using conventional FTIR spectroscopy (Fig. S2). The threefold improvement in performance makes this approach practically useful as a quantum-enabled vibrational spectroscopy technique. Although the onset of Rabi splitting occurs at a higher concentration than the theoretically calculated value of about 8.5 µmol/L owing to the suboptimal experimental conditions, this result experimentally confirmed the transition from weak to strong coupling for the platform. By plotting the Rabi splitting energy with respect to the MBN analyte concentration, we observed a strong linear correlation (Fig. 5F). This result demonstrates the feasibility of leveraging quantum vibropolaritonic systems as a quantitative sensing tool, where Rabi splitting serves as a unique and reliable transducer for quantifying analyte concentration. At higher analyte concentrations, where the Rabi splitting becomes more pronounced and particularly when the cavity has a small free spectral range (FSR), adjacent FP modes may spectrally overlap with the vibropolaritonic peaks, complicating spectral interpretation. This issue can be mitigated in future implementations using a shorter cavity length to increase the FSR, thereby reducing mode overlap and improving spectral clarity.

## DISCUSSION

It is important to know, however, that analyte concentrations studied in this proof-of-concept demonstration, ranging from 0.5 to 10 mmol/L, are higher than those typically encountered in practical molecular sensing applications. This observation reflects the fact that this quantum sensing platform has not yet been optimized for low-concentration sensing applications. Nevertheless, to our knowledge, this study represents the first successful demonstration of quantum vibropolaritonic sensing, marking a major milestone in using hybrid light-matter states for molecular analyses, including the capability to use concentration-dependent Rabi splitting. We believe that this study lays the foundation for quantum vibropolaritonic sensing by leveraging vibropolaritonic states achievable under ambient conditions as an exquisite quantum-enabled transducer for concentration-dependent molecular detection. This work establishes a paradigm for optical molecular analysis based on collective light-matter interactions. The difficulty in achieving VSC at lower concentrations in this initial study highlights the challenges inherent in this field, particularly in augmenting the cavity-molecule coupling strength. Nonetheless, the theoretical lower detection limit predicted by our theoretical analysis and FDTD simulations offers important clues in further improving the sensing performance. Specifically, future advances in cavity design (i.e., engineering smoother gold mirrors in an FP cavity with a higher quality factor), control and enhancement of molecular transition dipole orientation (e.g., enhancement through use of plasmonic arrays as artificial molecules), and strategies for suppressing solvent damping, could augment the total light-matter coupling strength, as recently demonstrated (*44*, *45*), and hold the key to fully unleash the potential of quantum vibropolaritonic sensing for practical quantitative analytical applications.

In summary, in this study, we first performed a systematic study of the feasibility and performance of quantum vibropolaritonic sensing through a combination of theoretical analysis and FDTD numerical simulations. Building on this theoretical foundation, we provided an experimental proof-of-concept demonstration of this quantum sensing strategy by coupling MBN molecules in DMSO with an FP cavity mode under VSC. Our results reveal distinct vibropolaritonic spectral features, which are fundamentally different from surface-enhanced Raman spectroscopy and cavity-enhanced absorption spectroscopy, and provide the first experimental demonstration of quantum vibropolaritonic sensing. We believe that this study marks a major milestone in harnessing hybrid light-matter states for molecular sensing. Through further innovations in cavity design and by exploring enhancement mechanisms to augment cavity-molecule coupling strength, we envision that the described quantum optical sensing strategy holds promise to impact application areas including chemical sensing, environmental monitoring, biomedical diagnostics, and bioprocess monitoring.

## MATERIALS AND METHODS

### Effective relative permittivity for molecular analytes in solution

The effective relative permittivity of the medium $\varepsilon_{\text{eff}}$ is modeled on the basis of the volume fraction–weighted mixing rule (*37*, *38*), given by

$$\varepsilon_{e}=\phi_{a}\cdot \varepsilon_{a}+\phi_{s}\cdot \varepsilon_{s}$$ (2)

where $\phi_{a}$ and $\phi_{s}$ are the volume fraction of the analyte and solvent with $\phi_{a}+\phi_{s}=1$ , $\varepsilon_{a}$ and $\varepsilon_{s}$ are their respective relative permittivity. Suppose that the analyte has a concentration of C(in mol/L) in the solvent with a molar volume $V_{m}\;\left(\text{in L/mol}\right)$ , its volume fraction can be calculated as $\phi_{a}=C\cdot V_{m}$ . The molar volume can be further obtained as $V_{m}=M/\rho$ , where ρ is the analyte density. Substituting $\phi_{a}$ into Eq. 2, we have

$$\varepsilon_{e}=C\cdot M/\rho \cdot \varepsilon_{a}+\left(1-C\cdot M/\rho \right)\cdot \varepsilon_{s}$$ (3)

As the solvent is not directly involved in the VSC, its relative permittivity $\varepsilon_{s}$ is modeled as a constant. The analyte is modeled as a Lorentz oscillator (*35*, *36*) with a frequency ω–dependent relative permittivity given by

$$\varepsilon_{a}\left(\lambda \right)=\varepsilon_{\infty }+\frac{F\omega_{0}^{2}}{\omega_{0}^{2}-\omega^{2}-i\gamma_{0}\omega }$$ (4)

where $\varepsilon_{\infty }$ is the analyte’s high-frequency (background) relative permittivity due to nonresonant contributions, $\omega_{0}$ is its vibrational transition energy with a damping rate of $\gamma_{0}$ with a unit of electron volts, F is the effective oscillator strength contributed by a large population of molecules collectively participating in the VSC with the FP cavity mode, and i is the imaginary unit. The effective oscillator strength F is dependent on the molecular concentration C and the effective mode volume $V_{\text{eff}}$ , given by $F=f_{0}\cdot \left(C\cdot N_{A}\right)\cdot V_{\text{eff}}$ , where $f_{0}$ is the intrinsic molecular oscillator strength and $N_{A}$ is the Avogadro’s number ( $N_{A}=6.022\times 10^{23}\;{\text{mol}}^{-1}$ ). Substituting F into Eq. 4, we have

$$\varepsilon_{a}\left(\lambda \right)=\varepsilon_{\infty }+\frac{f_{0}\cdot \left(C\cdot N_{A}\right)\cdot V_{\text{eff}}\cdot \omega_{0}^{2}}{\omega_{0}^{2}-\omega^{2}-i\gamma_{0}\omega }$$ (5)

The intrinsic oscillator strength $f_{0}$ is a dimensionless quantity that measures the probability of a transition between two quantum states (e.g., ground and excited states), which is given by

$$f_{0}=\frac{4\pi^{2}m_{e}}{3e^{2}}\omega_{0}^{2}\mu^{2}$$ (6)

where the electron mass is $m_{e}=9.11\times 10^{-31}\;\text{kg}$ , the electron charge is $e=1.60\times 10^{-19}C$ , and μ is the transition dipole moment. If we specifically consider the nitrile (─C≡N) stretching mode of 4-MBN with a vibrational transition at about $\omega_{0}=2250\;{\text{cm}}^{-1}$ and a transition dipole moment μ=0.1D , where $1\;D=3.335\times 10^{-30}\;C\cdot m$ , the intrinsic oscillator strength can be estimated and given by

$$f_{0}\approx 3\times 10^{-5}$$ (7)

Meanwhile, the effective mode volume $V_{\text{eff}}$ of an FP cavity can be estimated based on

$$V_{\text{eff}}=\frac{\pi \Omega_{0}^{2}L}{2}$$ (8)

Considering that the beam waist radius $\Omega_{0}=\sqrt{\lambda L/\pi }$ , where L is the cavity length, the effective mode volume can be rewritten as

$$V_{\text{eff}}=\frac{\lambda L^{2}}{2}$$ (9)

For an FP cavity made of a pair of gold mirror with a cavity length L=12μm at the vibrational transition frequency $\omega_{0}=2250\;{\text{cm}}^{-1}$ of 4-MBN, the effective mode volume is estimated to be

$$V_{\text{eff}}\approx 3.0\times 10^{-16}m^{3}$$ (10)

Now, if we consider that the solvent has a constant relative permittivity $\varepsilon_{s}=\varepsilon_{\infty }=2.13$ and given that M=135.19g/mol , $\rho =1.10\;g/{\text{cm}}^{3}$ , and $\gamma_{0}=8.76\times 10^{-4}\;\text{eV}$ , we can obtain the effective relative permittivity of the medium consisting of 4-MBN in DMSO as presented in Eq. 1.

### FDTD simulations

We performed FDTD simulations using Ansys Lumerical 2023 R2.1 (Anasys Inc., Vancouver, BC, Canada) software. For the studied FP cavity, only the gold mirrors and CaF_2_ IR window were modeled without considering the Cr and SiO_2_ layers. A Broadband Fixed Angle Source Technique plane wave was used to perform simulations of incident angle θ–dependent FTIR transmission spectra with periodic boundary conditions. Both the solvent permittivity and the high-frequency permittivity of the analyte were set to be 2.13. The relative permittivity of the analyte was modeled as a Lorentz oscillator, which was detailed above. The refractive index of CaF_2_ IR window was set to be 1.35. The dielectric function for gold was extracted from Johnson and Christy (*46*).

### Materials and chemicals

4-MBN, DMSO (suitable for high-performance liquid chromatography, ≥99.7%), the Specac Omni Cell demountable cell for IR spectroscopy, and the CaF_2_ IR windows were purchased from Sigma-Aldrich. Mylar films were purchased from Premier Lab Supply.

### Assembly of FP cavities

We used a Specac Omni Cell demountable cell to assemble an FP cavity for IR spectroscopy measurements. As the first step, a pair of gold mirrors was fabricated by successively depositing a layer of 2-nm Cr and 12-nm Au on CaF_2_ IR windows by e-beam evaporator. The gold mirrors were protected by depositing another layer of 300-nm SiO_2_ on top of the gold surface also by e-beam evaporator. Subsequently, the gold mirrors were assembled into the Specac Omni Cell demountable cell with the desired spacer thicknesses (i.e., 12 µm and 25 μm, respectively). By tightening or loosening using the screws, the cavity length can be fine-tuned to match the targeted vibrational transition frequencies.

### Characterizations

We used a Thermo Nicolet 6700 FTIR/near-IR spectrometer (refurbished from SpectraLab, Markham, ON, Canada) to take the transmission spectra and absorption spectra for detection of MBN in DMSO.
