# Supplementary material for: Quantum vibropolaritonic sensing
Source: Sci Adv. 2025 Aug 15;11(33):eady7670. doi: 10.1126/sciadv.ady7670 (PMC12356251; doi:10.1126/sciadv.ady7670)
Supplement: Supplementary file 1 — Figs. S1 and S2 [file sciadv.ady7670_sm.pdf]

Science Advances
AAAS

Supplementary Materials for

Quantum vibropolaritonic sensing

Peng Zheng *et al*.

Corresponding author: Ishan Barman, ibarman@jhu.edu; Peng Zheng, pzheng7@jhu.edu

Sci. Adv. **11**, eady7670 (2025)
DOI: 10.1126/sciadv.ady7670

This PDF file includes:

Figs. S1 and S2

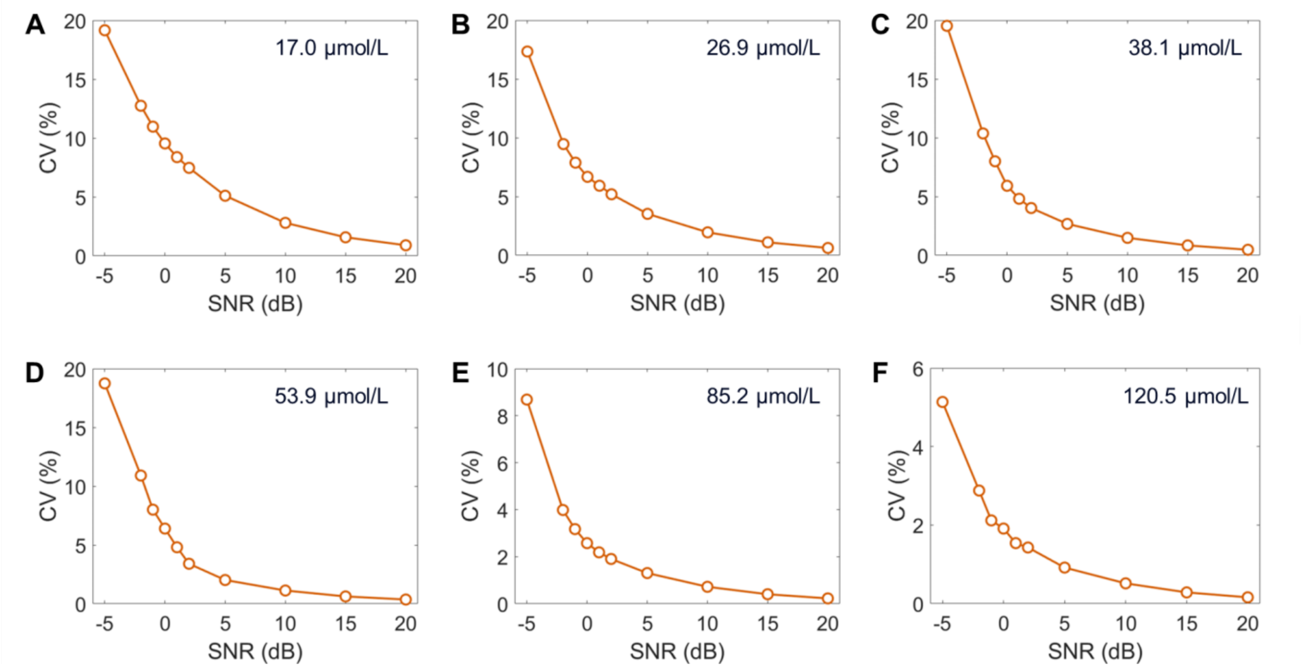

Fig. S1.

SNR-dependent CV for analytes with various concentrations as specified.

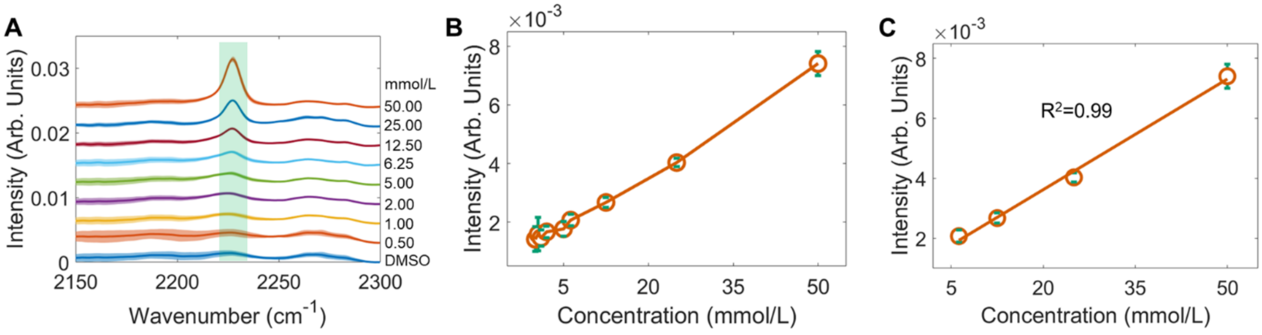

Fig. S2.

Conventional FTIR measurement of 4-MBN in DMSO. (**A**) Absorption spectral evolution of the characteristic nitrile group in MBN with increasing MBN concentration. The shaded regions, which are the vertical width of the spectral lines in each spectrum, represent the standard deviation, showing the degree of variation across multiple measurements (**B**) Intensity increase of the nitrile absorption peak in relation to the MBN concentration. (**C**) Linear regression analysis of the nitrile absorption peak intensity and the MBN concentration. For each concentration, the measurements were repeated three times. The error bars represent +/- 1 standard deviation for three measurements. In (**A**), the absorption spectra have been arbitrarily offset for clarity.
